# Draft Genome Sequences of Five *Carnobacterium* sp. Strains Isolated from Freshwater Ponds in Belgium

**DOI:** 10.1128/MRA.00955-19

**Published:** 2020-06-18

**Authors:** Konrad J. Korzeniowski, Shinjini Mukherjee, Caroline Souffreau

**Affiliations:** a Laboratory of Aquatic Ecology, Evolution and Conservation, KU Leuven, Leuven, Belgium; University of Southern California

## Abstract

Strains belonging to the genus *Carnobacterium* are Gram-positive bacteria that are widely distributed in the environment. Here, we report the draft genome sequences of five *Carnobacterium* strains isolated from freshwater ponds located in Flanders, Belgium, and sequenced on an Illumina HiSeq 4000 platform.

## ANNOUNCEMENT

Bacteria from the genus *Carnobacterium* have previously been isolated from various food sources (1–3), such as meat (4) and milk (5), and grow in a pH range of 5.5 to 9.1 (1, 3, 6). Fewer reports exist of isolates of this genus from aquatic environments (1, 6–8). To date, 11 species have been reported to belong to the genus (3, 4, 9–13). Here, we present draft genome sequences of five *Carnobacterium* sp. strains isolated from five different freshwater ponds located in Belgium within the scope of experimental work. These ponds were small (<1 ha), shallow (<3 m), and man-made. Table 1 includes the geographic coordinates of the ponds and some water quality variables.

**TABLE 1:** Genome sequence statistics summary, pond water quality characteristics, and pond geographic coordinates for the five isolated *Carnobacterium* strains

Variable	Data for *Carnobacterium* sp. 1290 strain:				
	PL12RED10	PL26RED25	PL17RED31	PL24RED07	PL17GRE32
Genome characteristics					
Total length (bp)	1,976,998	1,974,582	1,957,158	1,975,794	1,996,575
GC content (%)	39.34	39.46	39.48	39.46	39.42
No. of CDS^*a*^	1,812	1,741	1,800	1,790	1,832
No. of tRNAs	51	51	51	51	52
No. of rRNAs	4	3	3	3	3
No. of reads	6,095,916	6,238,674	5,210,544	5,894,490	6,301,572
Final coverage (%)	457.353	468.178	396.568	444.213	471.107
SRA accession no. for raw reads	SRX7894432	SRX7894431	SRX7894430	SRX7894429	SRX7894428
No. of scaffolds	43	72	42	71	49
Scaffold *N*_50_ (bp)	151,666	74,489	168,268	74,489	119,417
GenBank accession no. for assembled genomes	WVES00000000.1	WVEP00000000.1	WVEQ00000000.1	WVER00000000.1	WVEO00000000.1
Pond water quality data					
pH	10.14	7.16	6.93	8.91	7.39
Oxygen (mg/liter)	12.495	3.905	2.755	8.94	1.00
Chlorophyll-a (μg/liter)	72.87	59.52	97.92	219.04	41.90
Pond geographic coordinates	51°7′1.7796ʺN, 4°28′30.4392ʺE	51°10′18.39ʺN, 4°24′53.9496ʺE	50°59′27.6684ʺN, 3°59′50.0604ʺE	50°56′5.784ʺN, 4°51′30.8304ʺE	51°1′2.7984ʺN, 3°56′36.3012ʺE

a CDS, coding sequences.

Water (50 ml) from each pond was filtered using a 0.22-μm filter and cryopreserved in glycerol at −80°C before the isolates were cultured on King agar B medium at 24°C. For DNA isolation, five isolates were cultivated at 24°C on King agar B medium for 48 h. Bacterial DNA was isolated using a Qiagen DNeasy UltraClean microbial DNA extraction kit (Qiagen). Initial identification was done by sequencing the 16S rRNA gene using primers 27F and 1492R (14).

The DNA concentration of the samples was measured using a Quant-iT PicoGreen kit (Thermo Fisher Scientific); DNA integrity and purity were determined by electrophoresis (1% agarose gel with Midori Green [Nippon Genetics Europe]) and spectrophotometry (*A*_260_/*A*_280_; Infinite M Nano+ [Tecan]). Library preparation and sequencing were performed at BGI (Shenzen, China). DNA was fragmented using a Covaris ultrasonicator. Fragmented DNA was combined with end repair mix (Agilent, USA) and purified with an AxyPrep Mag PCR cleanup kit (Thermo Fisher Scientific). Adapters (adapter 1, GATCGGAAGAGCACACGTCTGAACTCCAGTCAC; adapter 2, AGATCGGAAGAGCGTCGTGTAGGGAAAGAGTGTA) were added, and adapter-ligated DNA was purified with an AxyPrep Mag PCR cleanup kit. Adapter-ligated fragments (insert size, 300 bp) were enriched by PCR amplification (95°C for 2 min; 6 cycles at 98°C for 15 s, 62°C for 30 s, and 72°C for 30s; 72°C for 5 min), and the PCR products were purified with an AxyPrep Mag PCR cleanup kit. Sequencing libraries were quantified using an Agilent Technologies 2100 bioanalyzer and an ABI StepOnePlus real-time PCR system. The resulting libraries (<800 bp) were sequenced on an Illumina HiSeq 4000 platform (150-bp paired ends) at BGI.

Trimmomatic v0.38 (15) was used for quality trimming of raw sequences with a Phred quality score cutoff of 33 and a minimum read length of 40. Unicycler v0.4.8 (16) software was used for *de novo* assembly of the genomes. Functional annotation and genome quality assessment were performed using the NCBI Prokaryotic Genome Annotation Pipeline (PGAP). Genome coverage was assessed with BBMap v38.72 (https://sourceforge.net/projects/bbmap/). The sequence statistics of the genomes are summarized in Table 1.

Pairwise comparison analysis using JSpeciesWS (17) (http://jspecies.ribohost.com/jspeciesws/) showed that all investigated strains belong to the same species (average nucleotide identity, >95%) and have highest similarity (>97.39%) to *Carnobacterium* sp. 1290. Based on ResFinder (https://cge.cbs.dtu.dk/services/ResFinder/) (18), no resistance genes were found. No intact phages were found using PHASTER (19) (http://phaster.ca/). Default parameters were used for all software.

### Data availability.

This genome sequencing project has been deposited in the NCBI Sequence Read Archive (SRA) under BioProject number PRJNA551600 and BioSample numbers SAMN12161540 to SAMN12161544. The accession numbers for the individual strains are given in Table 1.

## References

[1] Leisner JJ, Laursen BG, Prévost H, Drider D, Dalgaard P 2007 Carnobacterium: positive and negative effects in the environment and in foods. FEMS Microbiol Rev 31:592–613. doi:10.1111/j.1574-6976.2007.00080.x. 17696886 PMC2040187

[2] Leroi F, Matamoros S, Pilet MF, Gigout F, Pre H 2009 Selection and evaluation of seafood-borne psychrotrophic lactic acid bacteria as inhibitors of pathogenic and spoilage bacteria. Food Microbiol 26:638–644. doi:10.1016/j.fm.2009.04.011. 19527840

[3] Yost CK, Guan TY, Holley RA, Peirson M 2002 Carnobacterium viridans sp. nov., an alkaliphilic, facultative anaerobe isolated from refrigerated, vacuum-packed bologna sausage. Int J Syst Evol Microbiol 52:1881–1885. doi:10.1099/00207713-52-5-1881. 12361300

[4] Collins MD, Farrow JAE, Phillips BA, Ferusu S, Jones D 1987 Classification of Lactobacillus divergens, Lactobacillus piscicola, and some catalase-negative, asporogenous, rod-shaped bacteria from poultry in a new genus, Carnobacterium. Int J Syst Evol Microbiol 37:310–316. doi:10.1099/00207713-37-4-310.

[5] Mora D, Scarpellini M, Franzetti L, Colombo S, Galli A 2003 Reclassification of Lactobacillus maltaromicus (Miller et al. 1974) DSM 20342T and DSM 20344 and Carnobacterium piscicola (Collins et al. 1987) DSM 20730T and DSM 20722 as Carnobacterium maltaromaticum comb. nov. Int J Syst Evol Microbiol 53:675–678. doi:10.1099/ijs.0.02405-0. 12807185

[6] Nicholson WL, Krivushin K, Gilichinsky D, Schuerger AC 2013 Growth of Carnobacterium spp. from permafrost under low pressure, temperature, and anoxic atmosphere has implications for Earth microbes on Mars. Proc Natl Acad Sci U S A 110:666–671. doi:10.1073/pnas.1209793110. 23267097 PMC3545801

[7] Yanagida F, Chen Y-S, Yasaki M 2007 Isolation and characterization of lactic acid bacteria from lakes. J Basic Microbiol 47:184–190. doi:10.1002/jobm.200610237. 17440921

[8] Voget S, Klippel B, Daniel R, Antranikian G 2011 Complete genome sequence of Carnobacterium sp. 17-4. J Bacteriol 193:3403–3404. doi:10.1128/JB.05113-11. 21551290 PMC3133254

[9] Joborn A, Dorsch M, Olsson JC, Westerdahl A, Kjelleberg S 1999 Carnobacterium inhibens sp. nov., isolated from the intestine of Atlantic salmon (Salmo salar). Int J Syst Bacteriol 49:1891–1898. doi:10.1099/00207713-49-4-1891. 10555373

[10] Pikuta EV, Marsic D, Bej A, Tang J, Krader P, Hoover RB 2005 Carnobacterium pleistocenium sp. nov., a novel psychrotolerant, facultative anaerobe isolated from permafrost of the Fox Tunnel in Alaska. Int J Syst Evol Microbiol 55:473–478. doi:10.1099/ijs.0.63384-0. 15653921

[11] Franzmann PD, Höpfl P, Weiss N, Tindall BJ 1991 Psychrotrophic, lactic acid-producing bacteria from anoxic waters in Ace Lake, Antarctica; Carnobacterium funditum sp. nov. and Carnobacterium alterfunditum sp. nov. Arch Microbiol 156:255–262. doi:10.1007/BF00262994. 1793333

[12] Kim M-S, Roh SW, Nam Y-D, Yoon J-H, Bae J-W 2009 Carnobacterium jeotgali sp. nov., isolated from a Korean traditional fermented food. Int J Syst Evol Microbiol 59:3168–3171. doi:10.1099/ijs.0.010116-0. 19643884

[13] Snauwaert I, Hoste B, De Bruyne K, Peeters K, De Vuyst L, Willems A, Vandamme P 2013 Carnobacterium iners sp. nov., a psychrophilic, lactic acid-producing bacterium from the littoral zone of an Antarctic pond. Int J Syst Evol Microbiol 63:1370–1375. doi:10.1099/ijs.0.042861-0. 22798642

[14] Deng W, Wanapat M, Ma S, Chen J, Xi D, He T, Yang Z, Mao H 2007 Phylogenetic analysis of 16S rDNA sequences manifest rumen bacterial diversity in gayals (Bos frontalis) fed fresh bamboo leaves and twigs (Sinarumdinaria). Asian Australas J Anim Sci 20:1057–1066. doi:10.5713/ajas.2007.1057.

[15] Bolger AM, Lohse M, Usadel B 2014 Trimmomatic: a flexible trimmer for Illumina sequence data. Bioinformatics 30:2114–2120. doi:10.1093/bioinformatics/btu170. 24695404 PMC4103590

[16] Wick RR, Judd LM, Gorrie CL, Holt KE 2017 Unicycler: resolving bacterial genome assemblies from short and long sequencing reads. PLoS Comput Biol 13:e1005595. doi:10.1371/journal.pcbi.1005595. 28594827 PMC5481147

[17] Richter M, Rosselló-Móra R, Oliver Glöckner F, Peplies J 2016 JSpeciesWS: a Web server for prokaryotic species circumscription based on pairwise genome comparison. Bioinformatics 32:929–931. doi:10.1093/bioinformatics/btv681. 26576653 PMC5939971

[18] Zankari E 2014 Comparison of the Web tools ARG-ANNOT and ResFinder for detection of resistance genes in bacteria. Antimicrob Agents Chemother 58:4986. doi:10.1128/AAC.02620-14. 25028728 PMC4136053

[19] Arndt D, Grant JR, Marcu A, Sajed T, Pon A, Liang Y, Wishart DS 2016 PHASTER: a better, faster version of the PHAST phage search tool. Nucleic Acids Res 44:W16–W21. doi:10.1093/nar/gkw387. 27141966 PMC4987931

